# Changes in the spatial and temporal characteristics of China’s arid region in the background of ENSO

**DOI:** 10.1038/s41598-022-21712-4

**Published:** 2022-10-24

**Authors:** Zhanshuo Qi, Chenfeng Cui, Yutong Jiang, Yue Chen, Juanli Ju, Ning Guo

**Affiliations:** 1 College of Water Resources and Architecture Engineering, Northwest A&F University, Yangling, Shaanxi, 712100 China; 2 Key Laboratory of Agricultural Soil and Water Engineering in Arid and Semiarid Regions of Ministry of Education, Northwest A&F University, Yangling, Shaanxi, 712100 China; 3 College of Water Sciences, Beijing Normal University, Beijing, 100875, China; 4 College of Economics and Management, Northwest A&F University, Yangling, Shaanxi, 712100 China; 5 Institute of Surface-Earth System Science, School of Earth System Science, Tianjin University, Tianjin, 300072 China

**Keywords:** Hydrology, Hydrology

## Abstract

Arid regions are sensitive to changes in precipitation, while El Niño-Southern Oscillation strongly influences worldwide hydrometeorological processes. Temporal and spatial changes of characteristics including precipitation, annual mean temperature and area in China's arid region were analyzed, using daily precipitation and temperature data from 117 meteorological stations of 1961–2016. The results show that: (1) The arid region is getting warmer and wetter. During the past 56 years, the precipitation in the arid region have shown an increasing trend. The annual and quarterly precipitation all exist a cycle of about 4 years. There is a negative correlation between the area of the arid region and the annual mean temperature, which is significant at the 0.01 level. (2) The area of arid region has been on a downward trend since 1968, and there was a mutation in 1992. There are three main cycles of 24 years, 13 years and 5 years in the area of the arid region. During the study period, the spatial center of the arid region’s precipitation moved 0.14° to the north and 0.77° to the east. (3) The response of precipitation to ENSO is different between the eastern and the western arid region. El Niño events increased the area of China’s arid region in El Niño years and La Niña events increased the precipitation of China’s arid region in La Niña years. The response of China’s arid region to ENSO in the first half of the following year is opposite and the response in spring is the most significant. To sum up, in El Niño years the eastern arid region increased in area and precipitation, while in La Niña years the western arid region decreased in area and the eastern arid region increased in precipitation, which was related to the eastward movement of the spatial center of the precipitation.

## Introduction

The 200 mm isohyet is the dividing line between arid and semi-arid regions and it has changed a lot over the decades^1^. In this study, China’s arid region was defined as areas with annual precipitation less than 200 mm. In China, the area covered by the 200 mm isohyet includes most of Xinjiang, western Gansu, western Inner Mongolia, northern Qinghai and northwestern Tibet, accounting for about one-third of China's area. This arid region is situated in Eurasian continent’s hinterland. Besides, the terrain is complex and it is far from the ocean. It is one of the driest regions in the world, but there are a lot of snow-capped mountains distributed here ^2–4^. The runoff formed by the melt water from the snow-capped mountains provides a large amount of water resources for this area, forming the Tarim River, the largest inland river in China, and also provides the foundation for the local people's life and agricultural development^5,6^. In arid region, the precipitation is scarce and the ecology is fragile^7,8^, where lacking water resources is a major impediment to social and ecological process, as well as a threat to ecological security. The annual precipitation is one of the most important factors affecting industry and agriculture, and it is critical for long-term socio-economic growth^9^. Meanwhile, in the past few decades, China has carried out large-scale ecological restoration projects in arid and semi-arid regions, and studies have shown that changes in precipitation can have a significant effect on these artificial vegetation^10^.A large number of studies have shown that global climate change has had a huge impact on the spatial and temporal distribution of precipitation in China, and China’s arid region are sensitive to global climate change^11–13^. The precipitation in different regions of China has changed significantly different over the last few decades. The increase in precipitation in Northwest China is particularly obvious while there has been a slight decrease in precipitation across China^14,15^. However, the lack of water resources is still one of the main factors restricting local development, and it is of great significance to study water resources in this area^16–18^. Numerous studies in recent years have shown that this region and the entire northwestern China are gradually becoming warmer and wetter. Numerous studies have documented increased precipitation in many areas within this arid region^2,19^. This change has a huge positive impact on the ecological environment of the region. Complex climate change brings opportunities and challenges for future agricultural development and ecosystem in China’s arid region^17,20^.

The El Niño-Southern Oscillation (ENSO) is a signal of major inter-annual and decadal climatic change in the tropical Pacific air-sea system. ENSO has been proved to be a major contributor to global precipitation variability^21,22^. It influences precipitation in China via changing global atmospheric circulation^19^. The rise of precipitation in the northwest arid region has been proved to be intimately linked to the Siberian High, Western Pacific Subtropical High, and North American Subtropical High swings^23^. Besides, large-scale atmospheric circulation, such as ENSO, North Atlantic Oscillation (NAO) and Pacific Interdecadal Oscillation (PDO) have complicated interactions. In general, the climate in the arid regions of China is complex. Therefore, it is important to elucidate the influence of probably the most significant one of these factors. Previous studies on the variation characteristics have mostly focused on the analysis of the characteristics of precipitation amounts, precipitation days, precipitation intensity, and the extreme precipitation across watershed or provinces^24–27^. However, as an important basis for the division of arid regions: how have the 200 mm precipitation contour and various characteristics of the area changed? How does the response to ENSO differ within this region, which is far from the ocean and has a complex topography? Therefore, it is necessary to analyze the temporal and spatial variation characteristics of China's arid region and find the correlation between precipitation variation and ENSO. It is not only the need for regional weather and climate forecasting, but also the scientific basis for formulating national disaster prevention and mitigation policies.

## Results

### Temporal trends of characteristics of China’s arid region

**Figure 1:**
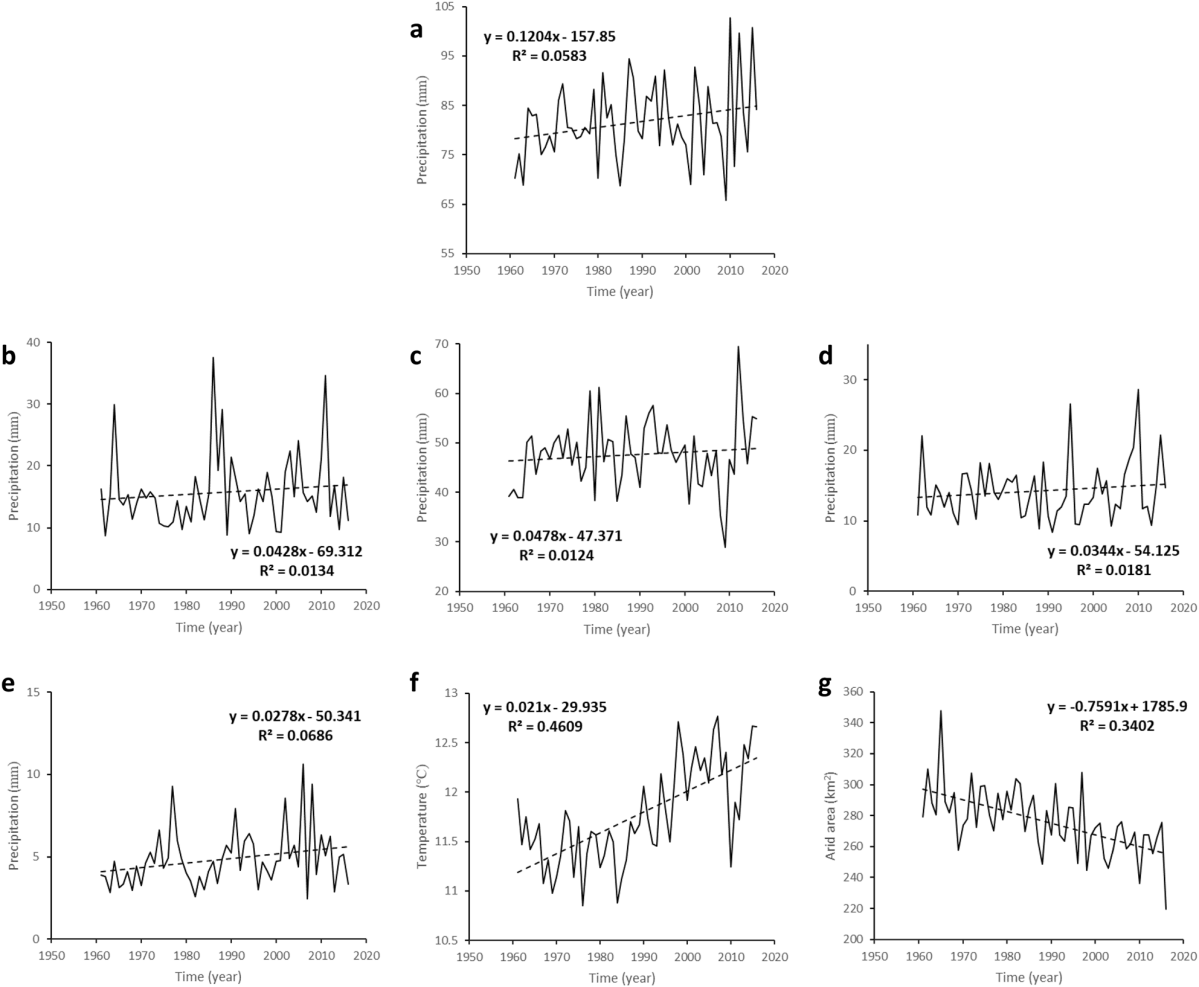
Change trends of annual and the seasonal precipitation, annual average temperature and area of China’s arid region during 1961–2016 (**a**) annual precipitation (**b**) spring precipitation (**c**) summer precipitation (**d**) autumn precipitation (**e**) winter precipitation (**f**) annual average temperature and (**g**) area of the arid region.

From Fig. 1a, it can be seen that the annual precipitation in the arid region ranges from 65.78 to 102.67 mm in the last 56 years, with an average of 81.57 mm, indicating a gradual variation and rising trend in the last 56 years. The linear regression result shows that its rising rate is 1.2 mm/10 years. In the past 10 years, the curve fluctuation has appeared extreme phenomenon many times, and the precipitation changes sharply between adjacent years. The precipitation in spring, summer, autumn and winter were range from 8.76–37.57 mm, 28.97–69.56 mm, 8.38–28.60 mm, and 2.45–10.61 mm respectively. The average precipitations are 15.71 mm, 47.59 mm, 14.20 mm and 4.84 mm respectively and their rising rates are 0.43 mm/10 years, 0.48 mm/10 years, 0.34 mm/10 years and 0.28 mm/10 years respectively. The annual average temperature change in arid regions is shown in Fig. 1f and its rising rate is 0.21 °C/10 years. Fig. 1g shows that the decreasing rate of the area is 7.59 × 10^4^ km^2^/10 years.

### Abrupt changes of characteristics of China’s arid region

**Figure 2:**
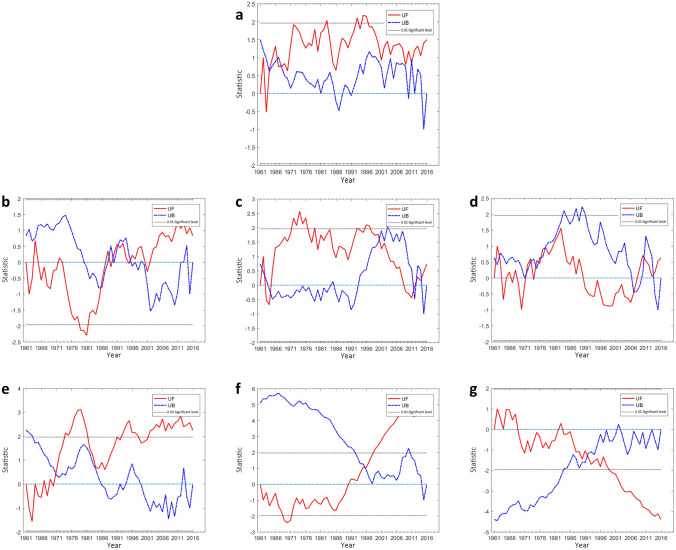
Mann—Kendall statistic curve of the annual and the seasonal precipitation sequence, annual average temperature sequence and area of the arid region sequence: (**a**) annual precipitation (**b**) spring precipitation (**c**) summer precipitation (**d**) autumn precipitation and (**e**) winter precipitation (**f**) annual average temperature and (**g**) area of the arid region.

The change points of the characteristics time series are presented in Fig. 2. From Fig. 2a, the M–K mutation test shows that the annual precipitation mutation points in arid region are predominantly focused in the first ten years, with the mutations occurring in 1963, 1966, 1968, and 2011. The UF and UB curves of multi-year precipitation have several intersections in spring, summer, and fall, while only one obvious intersection exists in winter. Figure. 2b represents the presence of mutations in spring precipitation, which occurred in 1988, 1990, 1992, and 1995. Figure. 2c suggests obvious mutations in summer precipitation, with the intersections in 1962, 1963, 1965, 2000, 2012 and 2013. Figure. 2d displays the intersections in autumn precipitation, with the intersections in 1962, 1972, 1973, 1976, 1978, 2007, 2010 and 2013. The obvious intersection in winter precipitation, occurred in 1971, can be detected in Fig. 2e followed by an increase after 1971.

It can be seen from Fig. 2f that the annual average temperature shows a downward trend from 1961 to 1990. It shows a significant annual average temperature decrease from 1968 to 1971 (*P* < 0.05) and suggests the presence of an intersection in the annual average temperature, which occurred in 1996, followed by a significant annual average temperature increase, after 1999 (*P* < 0.05). The obvious intersection in the area of the arid region, occurred in 1992, can be detected in Fig. 2g followed by an obvious decrease.

### The periodic law of Arid Region’s characteristics

The wavelet real-part contour map of precipitation, where the contour value represents the real part of the wavelet coefficient, primarily reflects the scale change of precipitation. The positive wavelet coefficient suggests heavy precipitation and the negative wavelet coefficient indicates light precipitation. The proportion of a certain time-scale in the entire time-series is represented by wavelet variance. The stronger the oscillation and the more substantial the periodic shift in the related time-scale, the higher the variance value. The less significant the periodic variation of the relevant time-scale, the smaller the variance value. The numerical modulus of the wavelet series shows the energy density at various timescales.

By using MATLAB to calculate wavelet coefficients, the periodic variation of precipitation in arid region was studied.

**Figure 3:**
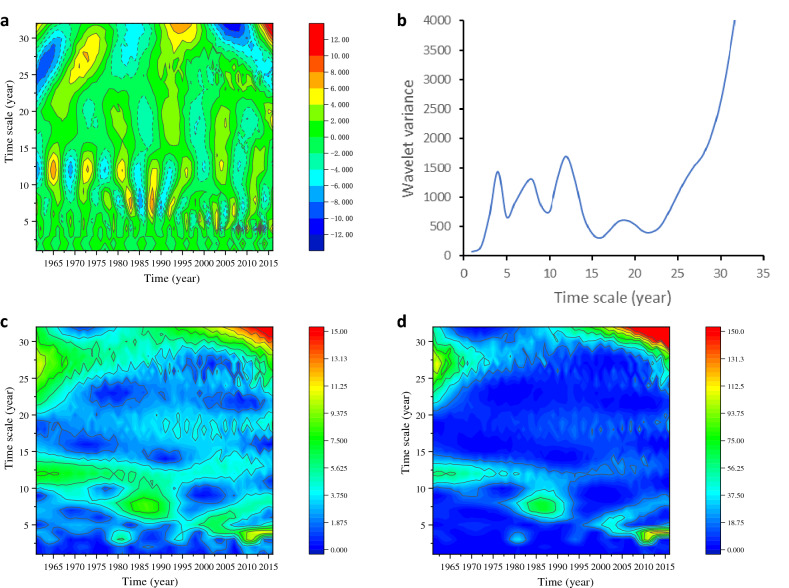
(**a**) Real contour map of the annual precipitation wavelet coefficients (**b**) Wavelet variance of the annual precipitation (**c**) Wavelet coefficient module contour map of the annual precipitation (**d**) Wavelet coefficient module square contour map of the annual precipitation.

As can be seen from Fig. 3a, there are various timescale structures in the precipitation series from 1961 to 2016, which are roughly 2a–4a, 5a–8a, 10a–14a, and 16–20a. The corresponding wavelet coefficients of 10a–14a are large, and the period is obvious. The annual precipitation wavelet variance Fig. 3b shows that there are four large peak points of annual precipitation, which indicates that there are four main cycles of annual precipitation. Among them, the four main cycles are 19a, 13a, 8a, and 4a. Among them, the cycle change period around 13a is the strongest, which is the first main cycle of annual precipitation in the arid region, and the fluctuation is more obvious between 1961 and 2008. However, the cycle of annual precipitation is gradually shortening. During 1985–1995, the most obvious cycle was 8a. After 1995, the most obvious cycle was 4a and the cycle of 13a almost disappeared. Besides, the cycle change period around 19a is not obvious during 1961–1990, but after 1990, the effect of this period is gradually obvious.

**Figure 4:**
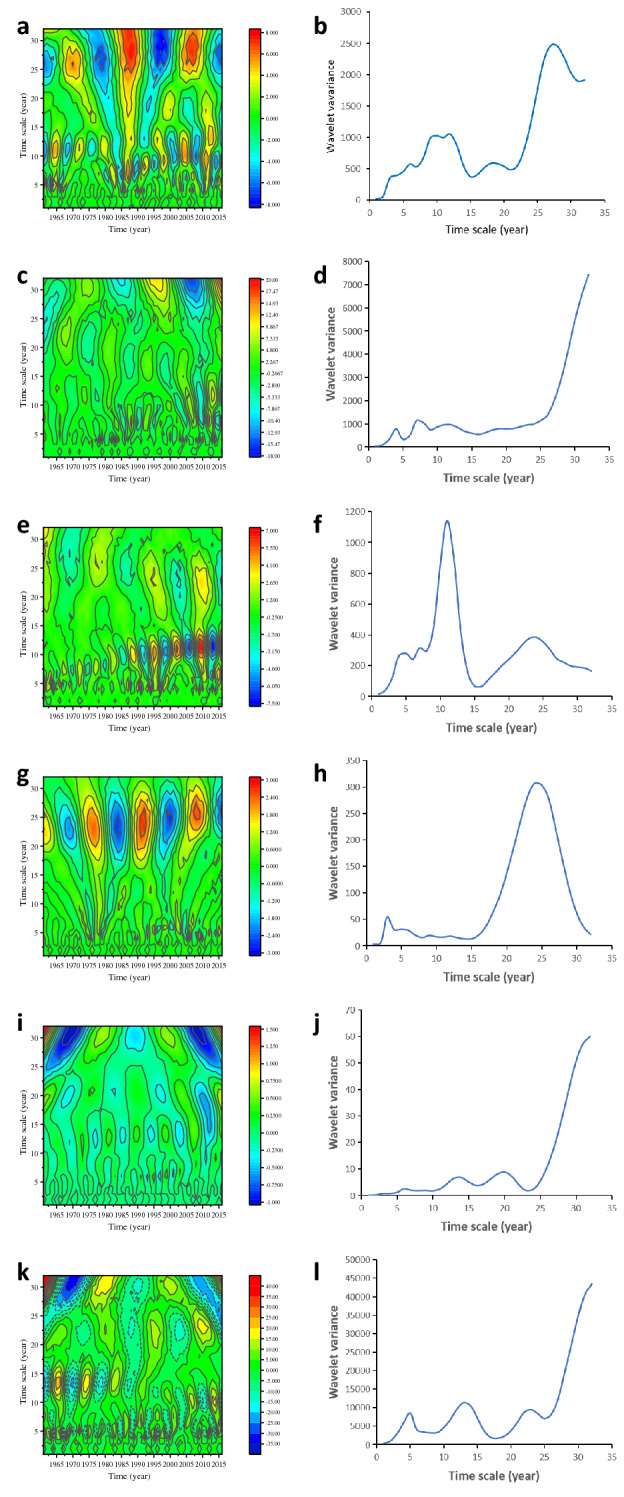
Real contour map of wavelet coefficients and wavelet variance of seasonal precipitation (**a**), (**b**) spring precipitation (**c**), (**d**) summer precipitation (**e**), (**f**) autumn precipitation (**g**),(**h**) winter precipitation (**i**),(**j**) annual average temperature (**k**),(**l**) area .

It can be seen from Fig. 4a that there are four main timescale structures, which are roughly 5–8 years, 10–13 years, 17–19 years, and 24–30 years. The corresponding wavelet coefficients of 24a–30a are large, and the period is obvious. From the wavelet variance diagram of spring precipitation (Fig. 4b), it can be seen that there are 5 main cycles of spring precipitation, which are 28a, 18a, 12a, 10a and 6a respectively. Among them, the period around 28a is the most obvious, which is the first main cycle of spring precipitation in the arid region, and the cycle change is the most obvious in the whole study period; 12a and 10a are the second and third main cycles of spring precipitation in the arid region, respectively. Among them, 12a mainly oscillated more obviously from 1961 to 1980, and the cycle change period around 10a from 2000 to 2016 was more obvious. The fourth and fifth major cycles correspond to the timescales of 18a and 6a, respectively, and the performance is not obvious. The fluctuations of the above five main cycles control the variation of spring precipitation in the arid region over the entire time domain.

Figure. 4c shows that the cyclical variation of summer precipitation is not obvious. The contours of the wavelet coefficients of summer precipitation in the arid region are relatively dense on the time scales of 3–9 years and 10–15 years. From the wavelet variance diagram of summer precipitation (Figure. 4d), it can be seen that there are three main cycles of 11a, 7a and 4a in the summer precipitation. Among them, the cycle change period of about 7a is the first main period of summer precipitation in the arid region, the second and third main periods correspond to the timescales of 11a and 4a, respectively, and the timescale of about 11a has experienced less than 2000 to 2016. The period is not obvious on the timescales of 11a and 4a in other time periods.

Figure. 4e shows that there are three main timescale structures, which are roughly 3–6 years, 8–11 years, and 21–26 years. In the range of 9–13 years, the frequency of high and low value center changes is the highest. From the wavelet variance diagram of autumn precipitation Figure. 4f, it can be seen that the four main periods of autumn precipitation in the arid region are 24a, 11a, 8a, and 4a respectively. Among them, the cycle change period around 11a is the strongest, which is the first main cycle of the autumn precipitation, and the cycle becomes more obvious after 1990. And the second main cycle is 24a, the fluctuation has always existed from 1961 to 2016 and gradually became stronger.

Figure. 4g shows that there are two main timescale structures, which are roughly 2–8 years and 20–27 years. In the range of 20–27 years, the center of high and low values changed significantly. From the wavelet variance diagram of winter precipitation (Figure. 4h), it can be seen that the three main cycle change periods of the winter precipitation are 25a, 6a, and 4a, respectively. Among them, the cycle change period around 25a is the strongest, which is the first main cycle of the winter precipitation, which lasted from 1961 to 2016 The timescale around 4a is the second main cycle, and the timescale of 6a shows a relatively high performance after 1990. Obviously, it is the third main cycle. The fluctuations of the above three main periods control the variation characteristics of winter precipitation in arid region in the whole time.

As can be seen from Figure. 4i, there are mainly two timescale structures, 10–15 years and 18–21 years. It can be seen from the wavelet variance of temperature (Figure. 4j) that 20a is the first main cycle of annual mean temperature, and 14a is the second main cycle. The timescale of 20a did not change significantly from 1980 to 1995, while the center of high and low values on the timescale of 14a had the highest frequency of change.

By using the Pearson correlation analysis, the relationship between the changes in the area of the arid region and the average annual temperature is significant at the level of 0.01. For every 1 °C increase in temperature, the area of the arid region decreases by 14.80 × 10^4^ km^2^. The annual precipitation shows a gentle upward trend with the average annual temperature.

From the Figure. 4k, there are three main timescale structures in the area series from 1961 to 2016, which are roughly of 3 to 7 years, 11 to 17 years, and 21 to 26 years. The corresponding wavelet coefficients of 11–17a are large, and the period is obvious. As can be seen from the wavelet variance of the area Figure. 4l, the three main cycles are 24a, 13a and 5a, of which the 24a period persisted from 1961 to 2016, and the 13a period only existed from 1961 to 1985.

### The spatial changing law of area of the arid region

**Figure 5:**
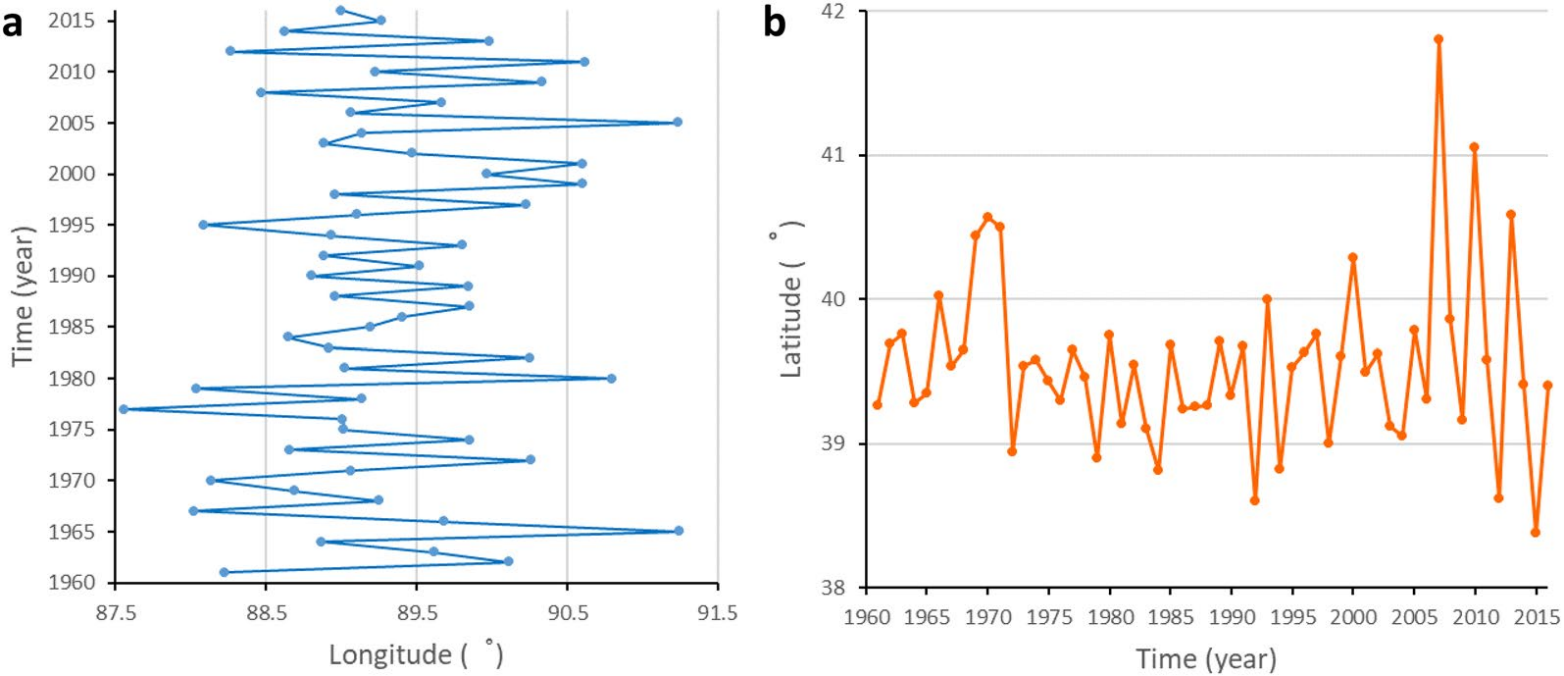
Longitudinal and latitudinal variations of the spatial center of the arid region’s precipitation (**a**) longitudinal variations and (**b**) latitudinal variations.

As the area of the arid region decreases, the spatial center of the arid region’s precipitation also moves. Using ArcGIS 10.5, the spatial center of the arid region’s precipitation is calculated from 1961 to 2016 and connected by a smooth curve, as shown in the Fig. 5.

According to the analysis, the spatial center of the arid region’s precipitation moved 1.30° to the north from 1961 to 1970, resulting in extreme east values of 91.24° E in 1965 and extreme west values of 88.02° E in 1967. The arid region's spatial center of precipitation moved 0.75 degrees to the south and 1.73 degrees to the east between 1971 and 1980. In the years 1971, 1972, 1977, 1979, and 1980, the spatial center of the arid region’s precipitation was in the shape of a five-pointed star. From 1981 to 1990, the spatial center of the arid region’s precipitation moved 0.20° to the north and 0.22° to the west, forming an extreme south value of 38.82° N in 1984 and an extreme east value of 90.25° N in 1982. From 1991 to 2000, the spatial center of the arid region’s precipitation moved 0.45° to the north and 0.61° to the east. From 1991 to 1994, the spatial center of the arid region’s precipitation swayed in the northeast-southwest direction. In 1999, the extreme east value of 90.60° E appeared, and in 1995, the extreme west value occurred. 88.09° E. From 2001 to 2010, the spatial center of the arid region’s precipitation moved to the northwest. In 2007, the extreme north value was 41.81° N, the extreme east value was 91.24° E in 2005, and the extreme west value was 88.47° E in 2008. From 2011 to 2016, the spatial center of the arid region’s precipitation mainly moved westward by 1.62°, in which the extreme north, extreme south, extreme east and extreme west values were generated in 2013, 2015, 2011 and 2012.

From 1961 to 2016, the spatial center of the arid region’s precipitation moved 0.14° to the north and 0.77° to the east, and the extreme north, extreme south, extreme east, and extreme west values appeared in 2007, 2015, 2005, and 1977. Although the area of the arid region is decreasing and the increased precipitation is beneficial to the industrial and agricultural production and future sustainable development of this region, the movement of the spatial center of the arid region’s precipitation indicates that the eastern areas adjacent to the arid region may be drier in the future.

### The relationship between the characteristics of arid region and ENSO

**Figure 6:**
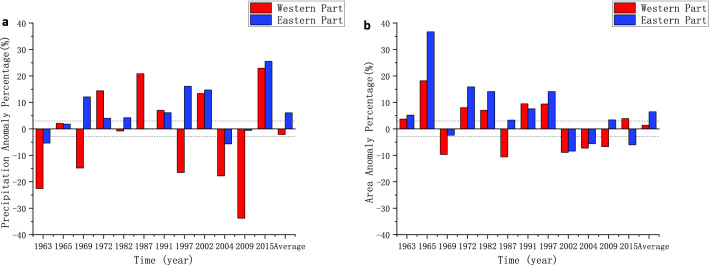
(**a**) Annual precipitation anomaly percentage and (**b**) area anomaly percentage in El Niño years.

The study found that the average area of the arid region in the past 56 years was 276.37 × 10^4^ km^2^, and the average annual precipitation was 81.57 mm. The average area of the arid region in the El Niño years was 286.06 × 10^4^ km^2^ and the average precipitation was 82.45 mm. The anomaly percentage of precipitation and area of eastern and western arid region in El Niño years were calculated respectively and the result is shown in Fig. 6. The areas of the western arid region and the eastern arid region in most El Niño years were greater than the average of their respective areas within 56 years. In 7 of the 12 El Niño years, the area of the western part is larger than the average, and in 8 of the 12 El Niño years the area in the eastern part is larger. The percentage of the area anomaly in the eastern part is larger than that in the western part in 10 years, indicating that the area in the eastern part has increased more. In 5 years of 12 El Niño years, the precipitation in the western arid region is obviously larger than the average, obviously smaller than the average in 5 years, and not significantly different from the average in 2 years. By using the linear regression method, the percentage of precipitation anomalies in the western arid region and the Niño 3.4 index were analyzed (R^2^ = 0.10), indicating that the linear relationship between the percentage of precipitation anomalies in the western arid region is not obvious. The precipitation in the eastern arid region was significantly larger than the average in 7 of the 12 El Niño years, significantly smaller than the average in 2 years, and not significantly different from the average in 3 years. The two years with less than average precipitation in the eastern arid region were 1963 and 2004, both of which were years with small Niño 3.4 index. By using the linear regression method, the percentage of precipitation anomalies in the eastern part and the Niño 3.4 index were analyzed (R^2^ is 0.70), and between the percentage of area anomalies and the Niño 3.4 index, R2 is 0.44. The same analysis was performed on the western arid region, and the correlations were not significant. The above results show that the El Niño events increase the area and the average precipitation of China’s arid region. In the western arid region, the correlation between precipitation and El Niño is not obvious. In the eastern arid region, both average precipitation and the area increased.

**Figure 7:**
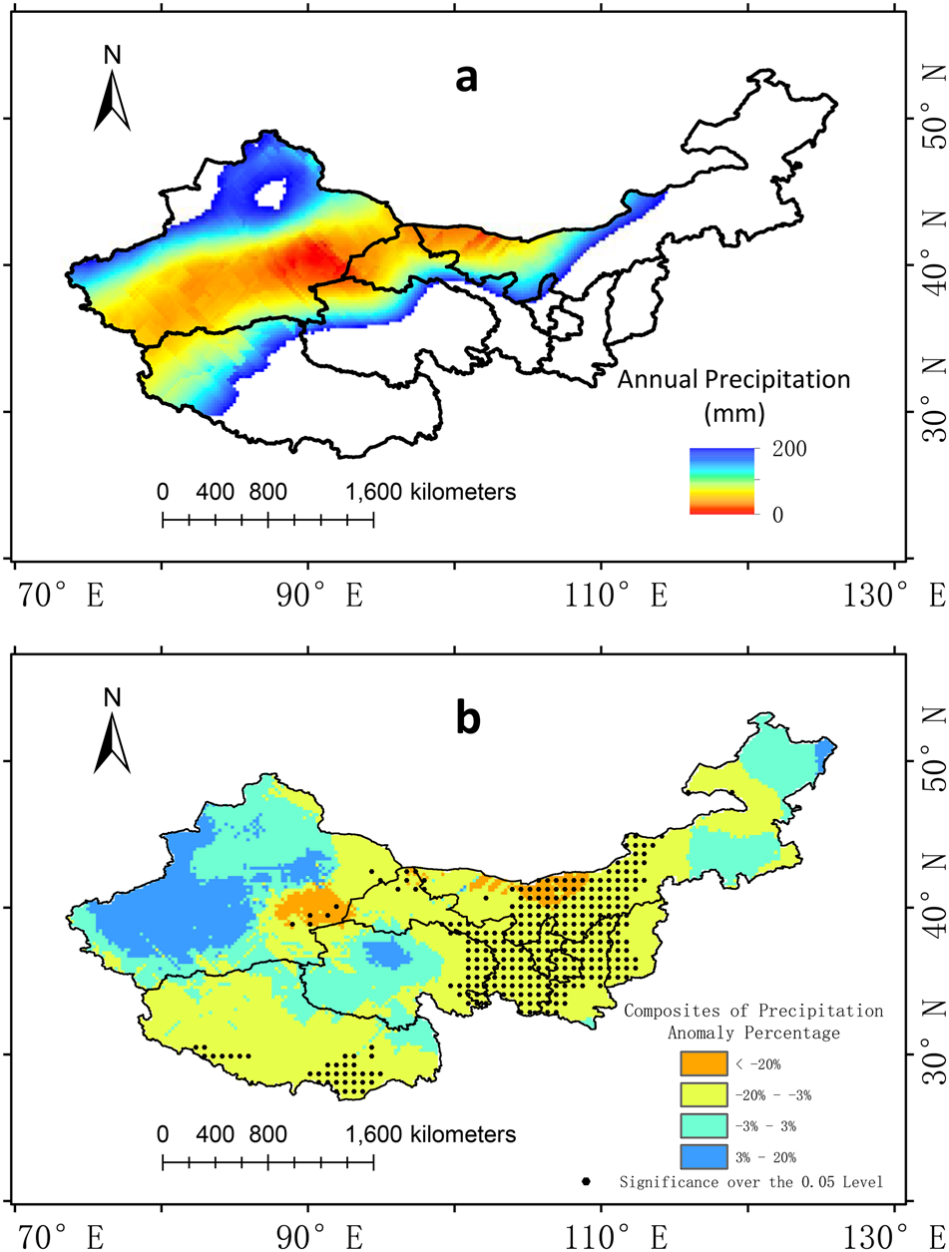
(**a**) The distribution of China’s arid region in El Niño years and (**b**) the composite annual precipitation anomaly percentage. The dotting represents the 0.05 significance level. The maps are generated with Arc Map version 10.5 (http://www.esri.com/sofware/arcgis).

Figure. 7a shows the distribution of the 200 mm isohyet in composited El Niño years and Fig. 7b shows the composite anomaly distribution of precipitation in Northwest China. The picture shows that the western part of Inner Mongolia, which is also the eastern arid region, experienced a significant decrease in precipitation in El Niño years. In the western arid region, most part of Xinjiang showed an increase in precipitation without passing the significant test. Eastern part of Xinjiang showed a decrease trend and parts of them passed the significant test. The result is consistent with the above analysis of El Niño's less significant effect in the western arid region compared to the eastern part.

**Figure 8:**
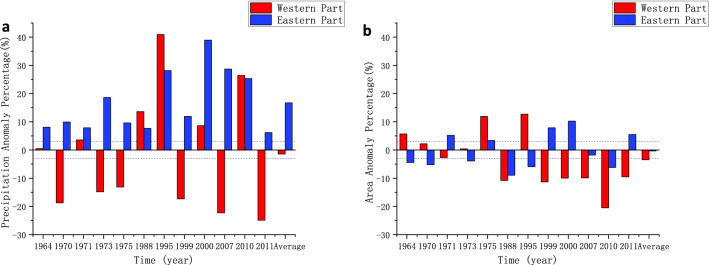
(**a**) Annual precipitation anomaly percentage and (**b**) area anomaly percentage in La Niña years.

The average precipitation in La Niña years was 83.026 mm, and the average area of the arid region was 262.180 × 10^4^ km^2^. The La Niña event increased the average precipitation in the arid region and reduced the area of the arid region. The anomaly percentage of precipitation and area of eastern and western arid region in La Niña years were calculated respectively and the result is shown in Fig. 8. In 6 of the 12 La Niña years, the area of the western and eastern arid region is smaller than the average. The precipitation in the western arid region is significantly smaller than the average in 6 years, and is bigger in 4 years. In the eastern arid region, the precipitation is bigger than the average in all 12 La Niña years. By using the linear regression method, the percentage of precipitation anomalies in the eastern part and the Niño 3.4 index were analyzed (R^2^ = 0.58), and between the percentage of area anomalies and the Niño 3.4 in10dex, R^2^ is 0.73. It indicates that the precipitation and area in the eastern arid region are related to La Niña. The same analysis was performed on the western arid region, and the correlations were not significant. Above results show that the La Niña events correlated with reduction in the area of the arid region, and the overall average precipitation in arid region increases more than that in El Niño years. In La Niña years, the average precipitation in the eastern arid region increases, and the area decreases.

**Figure 9:**
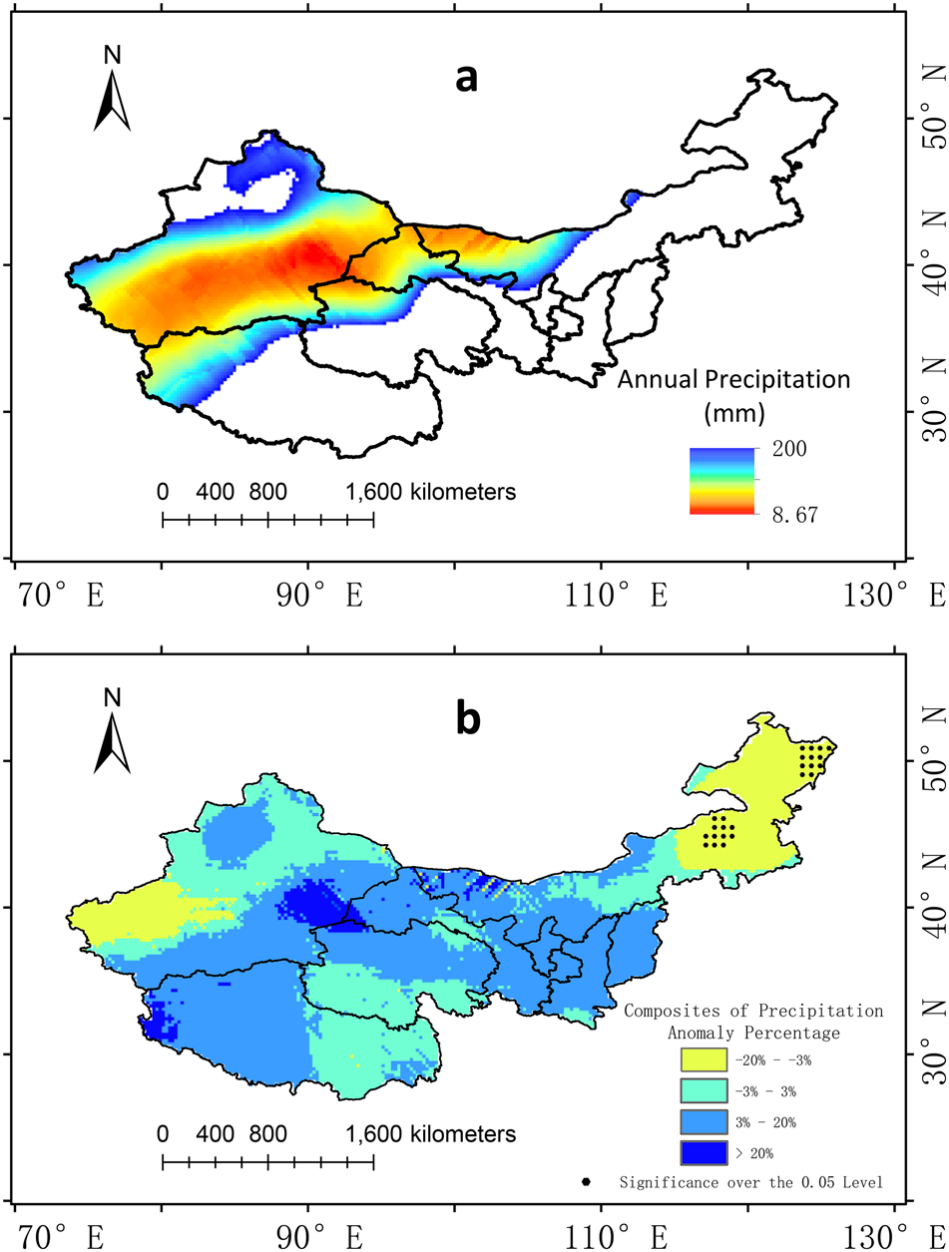
(**a**) The distribution of China’s arid region in La Niña years and (**b**) the composite annual precipitation anomaly percentage. The dotting represents the 0.05 significance level. The maps are generated with Arc Map version 10.5 (http://www.esri.com/sofware/arcgis).

Figure. 9 shows that La Niña events have a completely different impact on the western arid region. In La Niña years, widespread and obvious increase in precipitation appeared in most part of the arid region. Compared with El Niño years, the arid region in La Niña years is slightly smaller. However, almost no part in the arid region passed the significance test. This result is consistent with the above-mentioned insignificant correlation with Niño 3.4 index.

### The delayed impact of ENSO on Northwest China

**Figure 10:**
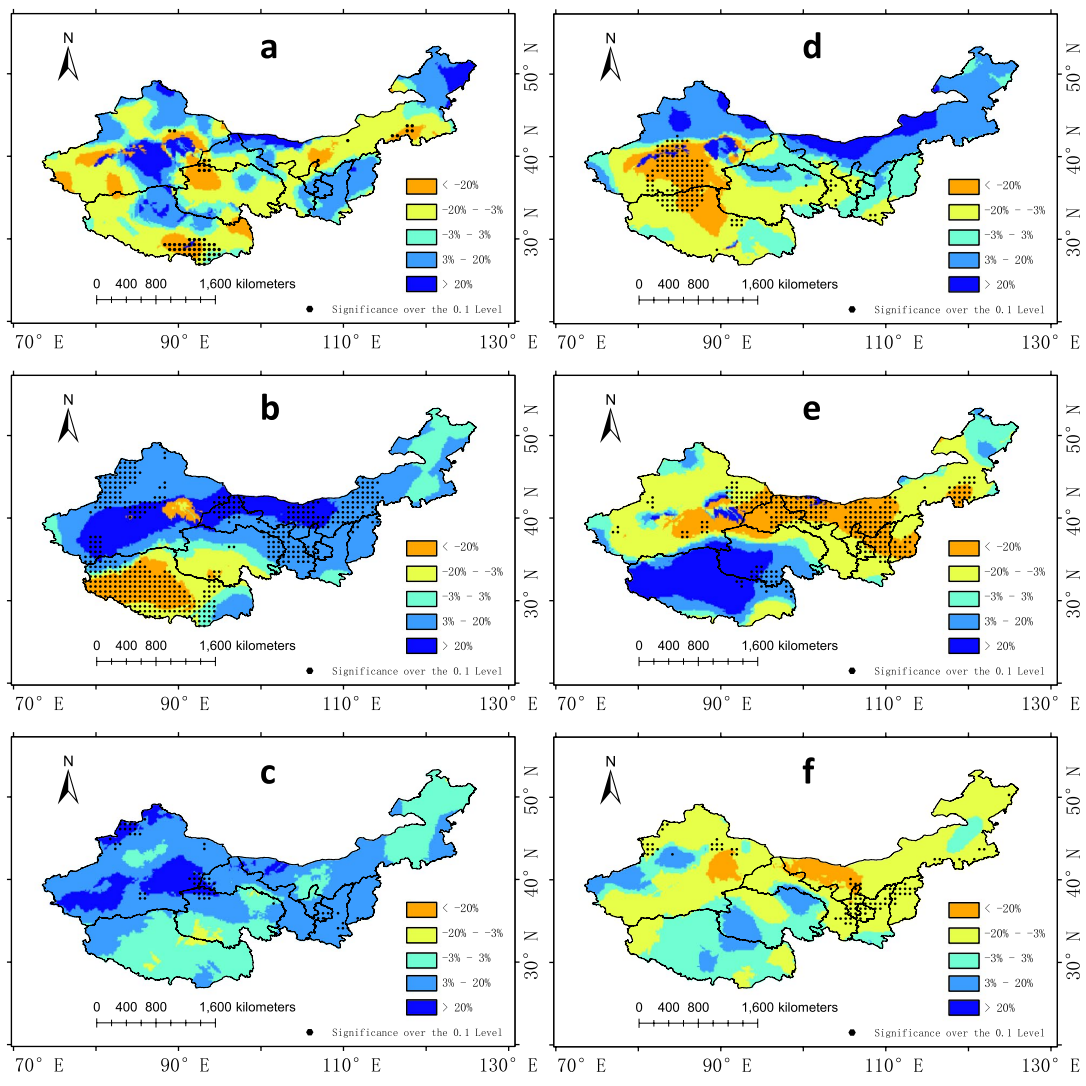
The composite seasonal precipitation anomaly percentage for D(0)JF(1), MAM(1), and JJA(1) seasons. Figures (**a**–**c**) represent El Niño years and Figures (**d**–**f**) represent La Niña years. The dotting represents the 0.1 significance level. The maps are generated with Arc Map version 10.5 (http://www.esri.com/sofware/arcgis).

ENSO's impact on China may be delayed^28^. In the selected representative years, Niño 3.4 in D(0)JF(1) all meet the criteria of an El Niño event or a La Niña event. The precipitation anomalies of D(0)JF(1), MAM(1), and JJA(1) seasons were analyzed and their significance was calculated. The results are shown in the Fig. 10.

Figure. 10a,b,c shows the delayed impact of El Niño events. From the figure, it can be seen that the precipitation in the following year of El Niño has the opposite change with that in El Niño years. Although in D(0)JF(1) most of Northwest China is still dominated by precipitation reduction, in MAM(1) the area where precipitation increases widely with a significance level of 0.1 is more than the other two seasons. In spring, MAM(1), most of the arid region showed a significant increase in precipitation except Tibet, which still had a significant decrease. In summer, precipitation in northwest China generally increased, although only a small part of the region passed the significance test.

Figure. 10d,e,f shows the delayed impact of La Niña events. The cumulative anomaly percentage plot def shows that, as in the case of El Niño, the precipitation in the following year changes in the opposite direction to that in the current year, and at MAM(1), a large area has a significance level of 0.1. In D(0)JF(1), the increase in precipitation mainly occurs in the northern region. In MAM(1), there was a significant decrease in precipitation in the arid region, mainly in Inner Mongolia and northern Gansu.

## Discussion

### Impact of arid region’s changes on supply–demand relationships of water resources

The arid and semi-arid regions in Northwest China has shown a warming-wetting trend, which is different from the trend of ‘dry-get-drier and wet-get-wetter’ in most other areas of the world19. Changes in various characteristics from 1961 to 2016 in the arid region has been studied, including most of Xinjiang, western Gansu, western Inner Mongolia, northern Qinghai and northwestern Tibet. In our research, the annual precipitation has experienced continuous growth, and the linear growth rate of summer precipitation is the highest, which is the same as previous researches on Xinjiang precipitation^28^.

Using wavelet periodic analysis method to further study the periodic law, we found that from 1961 to 2016, the three periods of the area were 24a, 13a and 5a, and 13a is the first main cycle. For the annual mean temperature in the arid region, the first main cycle is 20a and it was not significant from 1980 to 1995. In 2016, the annual mean temperature was in the middle of rising phase. There were four main cycles of 19a, 13a, 8a, and 4a in the annual precipitation in arid region and the obvious cycle of 13a existed from 1961 to 2008. Besides, the result also shows that the cycle of annual precipitation is gradually shortening. During 1961–1985, the only obvious cycle was 13a. However, during 1985–1995, the most obvious cycle was 8a. After 1995, the most obvious cycle was 4a and the cycle of 13a almost disappeared. This periodic variation in precipitation may be related to solar activity^19^,^30^. Although the cycle around 19a was relatively insignificant in the four time periods, it existed for the longest time and was getting more significant. Choosing 19a as the cycle, the precipitation was in the middle of rising phase in 2016. The annual mean temperature is also in the cycle of rising phase. According to five global climate models provided by ISI-MIP, Jun Yin et al. predicted that the annual precipitation in Northwest China may decrease from 2011 to 2050^31^. This matches our wavelet periodic analysis results.

Due to lack of water resources, China's arid region is typical arid ecologically fragile area^32^. Water has always been one of the main factors limiting the local development^33^. Agriculture in arid regions relies on irrigation, which is also the largest water consumption in the arid region and it is highly dependent on runoff from snow/glacier-fed meltwater. Supply–demand relationships of water resources have been alleviated in some parts of arid regions thanks to the current increase in precipitation and runoff, and the improvement of water-saving agriculture^34^. Studies have also shown that the increase in runoff in arid regions cannot simply be attributed to the increase in snow/glacier-fed meltwater And increased rainfall contributes more to the increase in runoff in some rivers6. In addition, our results show that the area of the arid region is decreasing at a rate of 7.59 × 10^4^ km^2^/10 years and the area decreased by 59.87 × 10^4^ km^2^ between 1961 and 2016. This means that more areas may have less water stress for agricultural irrigation. From the perspective of spatial distribution, these reduced arid regions are mainly in northern Xinjiang, which is also consistent with the research results of the reduction of extreme drought in northern Xinjiang in recent years18. But changes in arid region also pose challenges for water management^35^. Rising temperatures in arid regions could exacerbate the conflict between the supply and demand for water resources. Although the current increase in temperature has accelerated the melting of glaciers and produced more meltwater for agricultural irrigation, the decrease of meltwater from glaciers in the future may bring a crisis to agriculture in arid regions^16^. Without proper policy management of land and water resources, oases can easily be degraded. This phenomenon has already occurred in some oases in many arid countries and regions^36^. In conclusion, our study shows that the characteristics of precipitation in China's arid regions have undergone a series of sustained and significant changes in recent decades, and how water resources management in China's arid regions responds to these changes deserves more in-depth research.

Besides, changes in the distribution of precipitation and drought in Northwest China have been noted in some studies^13^,^29^,^37^. In our study, the spatial center of precipitation in the arid region is used to analyze the changes, and the results show that the longitude of the spatial center of precipitation in the arid region presents a complex and continuous periodic change, while the latitude of the spatial center of precipitation in the arid region is relatively stable before 2000 and then began to fluctuate greatly. The longitude of the precipitation center in the arid region moves eastward at a rate of 0.07°/10 years. Although there is no obvious trend in the linear regression of the latitude of the spatial center in the arid region, it can be clearly seen from the spatial distribution that Northern Xinjiang, which originally belonged to the northwest of the arid region, has been transformed from an arid region to a semi-arid region. In the east of the arid region, the arid region has expanded to the northeast. Benefiting from the current trend of warming and humidification, the area of the arid region is decreasing more obviously than its eastward expansion trend, but it is necessary to pay attention to the movement of the arid region in the future.

### Temporal and spatial differences of ENSO’s effects and the possible mechanisms

The arid regions of China are affected by the large-scale atmospheric circulation, and the combined factor that has the greatest impact on precipitation is ENSO-NAO^38^. According to the Niño 3.4 index, we selected the most significant El Niño years and La Niña years during 1961–2016, 12 years each. In El Niño years, the area of the eastern arid region increased. This area is influenced by the East Asian monsoon, and the expansion of the arid region may be due to the weakening of the monsoon by El Niño. Analysis of the composite precipitation anomaly percentage shows the large-scale precipitation reduction in the eastern part of the arid region. In the western part of the arid region, the precipitation increased slightly and did not pass the significance test. This may be due to the complex topography in the western part of the arid region, and different terrains in the region have different responses to El Niño and High-altitude mountains are conducive to the formation of precipitation by atmospheric water vapor. Some studies have shown that part of the water vapor in this region comes from the North Atlantic and Indian Oceans, so the western part of the arid region may be more affected by the western North Pacific subtropical high, the Indian summer monsoon, NAO and other factors^39^. In addition, analysis of the delayed effects of El Niño shows that the response of Northwest China to El Niño in the following spring, MAM (1), is the most obvious. The following spring precipitation showed a large-scale and significant increase trend, and this increasing trend continued into summer.

For La Niña years, precipitation in the eastern arid region was above average in all 12 La Niña years, while precipitation in the western part was less than average in 7 of 12 years. The area of the eastern arid region did not increase or decrease steadily during La Niña years, while the area of the western arid region decreased significantly over 7 of 12 years. Analysis of the composite precipitation anomaly percentage shows the large-scale precipitation increase in the arid region. The reduction of the area of the arid region is mainly due to the increase of precipitation in the northern part of Xinjiang, which changes to the semi-arid region. The arid region showed an overall increase in precipitation in La Niña years. The arid region showed an overall increase in precipitation in La Niña years. However, as in El Niño years, the analysis of the delayed effects showed an opposite widespread decrease in precipitation in the latter years of La Niña years. Probable cause is that La Niña enhanced the Indian High, causing the Somali cross-equatorial flow^12^. Our research shows that the spatial center of precipitation in the arid region exhibits interdecadal periodic fluctuations in the latitude direction, and the fluctuation in the longitude direction has intensified in recent years. The relationship between these phenomena and global climate change needs to be further studied. Analysis of the effects of El Niño and La Niña on arid regions reveals how ENSO affects the area, but as other studies have mentioned, the global environmental factors are not simply linear in their impact on arid regions^39^. In order to better explain the mechanism that affects precipitation in arid regions, it is necessary to consider a variety of factors for a more detailed regional division of arid regions, and to comprehensively consider the interaction of each factor. Figure. 7,9 provide a reference for the further division of regions in arid regions when studying the influence of atmospheric circulation in arid regions.

## Conclusions

In this study, linear regression method, spatial variability analysis, M–K mutation test, and wavelet periodic analysis were used to analyze the changes of the characteristics of precipitation, area and annual average temperature in China's arid region from 1961 to 2016. The impact of El Niño events and La Niña events on precipitation in China’s arid region was analyzed, and reached the following conclusions:

1. From 1961 to 2016, China’s arid region experienced a warm-wet climate regime. The annual and quarterly precipitation in the arid region showed different degrees of growth. There is a cycle of 4 years in the annual and quarterly precipitation, but the significance is different. There are four main cycles of 19a, 13a, 8a, and 4a in the annual precipitation in arid region, and there is an obvious cycle of 13a from 1961 to 2008. The annual precipitation cycles of 19a and 4a gradually become obvious. The first main cycle of spring, summer, autumn and winter are respectively 28a, 7a, 11a, and 25a. In 1990, the annual average temperature in arid region changed from a decreasing trend to an increasing trend. The first main cycle of the annual mean temperature is 20a.
2. The area of arid region has been on a downward trend since 1968, and there was a mutation in 1992. The three area cycles are 24a, 13a and 5a. In 2016, the area of arid region was in the declining phase of the cycle. The area of the arid region is decreasing at a rate of 7.59 × 10^4^ km^2^/10 years and the area decreased by 59.87 × 10^4^ km^2^ between 1961 and 2016. The area of arid region is negatively correlated with the annual mean temperature, and the correlation is significant at the 0.01 level. The shrinking of the arid region means more precipitation in more areas, which has positive implications for industrial and agricultural production in these areas. In addition, from 1961 to 2016, the spatial center of precipitation in the arid region moved 0.77° to the east.
3. The response of precipitation to ENSO is different between the eastern and the western arid region. El Niño events increased the area of arid region in China in El Niño years. The average precipitation in the arid region increased slightly, and the area of the eastern arid region increased more than the western arid region. The response of China’s arid region to El Niño in the following spring, MAM (1), is the most obvious. There was a widespread decrease in precipitation in the arid region in El Niño years, but an increase in precipitation in the spring and summer of the following year. To the opposite, La Niña events increased the precipitation of China’s arid region. There was a widespread increase in precipitation in La Niña years, but a decrease in precipitation in the spring and summer of the following year. To sum up, in El Niño years the eastern arid region increased in area and precipitation, while in La Niña years the western arid region decreased in area and the eastern arid region increased in precipitation, which was related to the eastward movement of the spatial center of the precipitation.

## Data and methods

### Study area description

**Figure 11:**
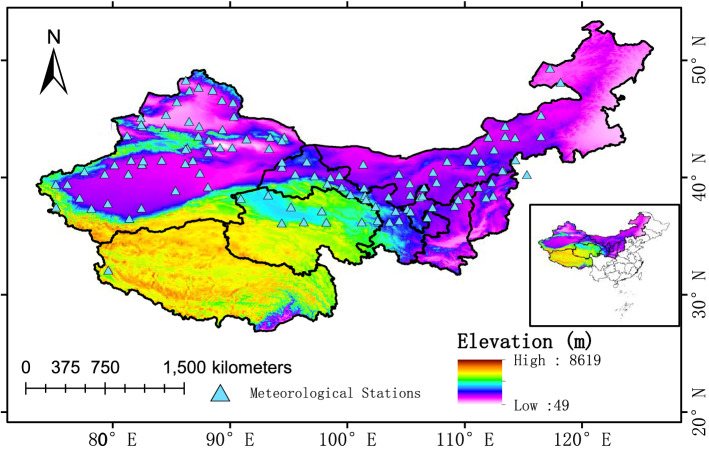
The locations of meteorological stations in arid region of China The map is generated with Arc Map version 10.5 (http://www.esri.com/sofware/arcgis).

The area of China's arid region (Fig. 11) mainly includes the Uygur Autonomous Region of Xinjiang, the northern Qinghai Province, the west of Inner Mongolia, and the Gansu Province^40^. As This region is situated in the interior of the Eurasian continent, away from the ocean, and is therefore less affected by the summer monsoon. Although domestic and international research have different views on the definition of arid region, they all agree that water is the most significant constraint to arid region development. The precipitation is the main influencing factor of water resources in arid region. Since the precipitation in Northwest China has changed significantly in the past 60 years, this study took the area with annual precipitation below 200 mm as the arid region to analyze its characteristic changes.

### Determination of ENSO Years

The El Niño–Southern Oscillation (ENSO) is a climatic phenomenon induced by the interplay of the atmosphere and the ocean across the tropical eastern Pacific Ocean^41,42^. It is a cyclic phenomenon with a period of 2 to 7 years that occurs around the equatorial Pacific Ocean and is acknowledged as the strongest signal of interannual climate variability. Their influence extends beyond low latitudes around the equator, affecting medium and high latitudes as well. ENSO redistributes heat and water vapor by altering large-scale air flow at the surface and small-scale ocean circulation^43^, and then has a significant impact on the temperature and precipitation in the middle and low latitudes. Some studies show that in El Niño years, the precipitation in China is relatively low^44,45^, and droughts are more likely to occur in North China (especially the Yellow River Basin) and northwest regions^9,46^; while in La Niña years, the northwest region is prone to heavy precipitation due to heavy precipitation and the probability of flood disasters has increased^47,48^. In this study, the ENSO year is determined according to the standard proposed by Barnston et al^49^. For this standard, El Niño years have a monthly average of the Niño 3.4 index from July to October > 0.5, whereas La Niña years have a monthly average of less than or equal to − 0.5. The only exception is 1985, because the La Niña event occurred in the first half of the year and its monthly average of the Niño 3.4 index for the whole year was still less than − 0.5. As indicated in Table. 1, 12 El Niño years and 12 La Niña years are chosen out from 1961 to 2016.

### Historical Data

The climate data from 1961 to 2016 was taken from the China Meteorological Administrator's website (http://cdc.cma.gov.cn/), which provided monthly temperature and precipitation. Considering the data in this study must meet the requirements of accuracy and completeness, the data of 692 stations with no missing measurements in the study area are selected for the study.

### Dynamic arid region

Annual precipitation and quarterly precipitation are calculated by Thiessen Polygons and the Kriging spatial interpolation tool of ArcGIS is used on the precipitation data. After using the ordinary kriging interpolation method^50^, the area with annual precipitation less than 200 mm is regarded as arid region, so the data from 117 stations during 1961 and 2016 is chosen in the end. The Thiessen Polygons Method is also called the weighted average method. Using the Thiessen polygons calculation tool in ArcGIS 10.5, we can directly calculate the area precipitation of the watershed and the operation is simple^51^. The precipitation intensity in the polygon area is represented by the precipitation of the unique meteorological station contained in the polygon, and the surface average precipitation is calculated according to the sum of the area weights occupied by each polygon and the precipitation of the meteorological station. This method is repeated for the data in every time period to obtain the dynamic arid region due to the change of precipitation distribution over time. Using this method to calculate precipitation is more accurate than the arithmetic average method, and it is more conducive to reflect the trend of changes in arid region.

### Mann—Kendall (M–K) mutation test

The Mann—Kendall test is used in this study to discover monotonic trends in the data series under consideration since it is more powerful than ordinary parametric trend tests for non-normally distributed series. The computation is also straightforward^52^. For a time series x:

1 $$S_{k} = \mathop \sum \limits_{i - 1}^{k} r_{i} { }\left( {k = 2,3,{ } \ldots ,{ }n} \right)$$

The value of *r*_*i*_ in the formula is as follows:

2 $$r_{i} = \left\{ {\begin{array}{*{20}c} {1 x_{i} > x_{j} } \\ {0 x_{i} \le x_{j} } \\ \end{array} { }j = 1,2, \ldots ,i} \right.$$

Assume that the time series x is randomly independent and define the statistics:

3 $$UK_{k} = \frac{{\left| {s_{k} - E(s_{k} )} \right|}}{{\sqrt {{\text{var}} (s_{k} )} }},\;k = 1,2, \ldots ,n$$

When the *n* variables of the series x_1_, x_2_, $\cdots$ , x_n_ are independent of each other and have the same continuous distribution, $E\left( {s_{k} } \right)$ and $Var\left( {s_{k} } \right)$ are as follows:

4 $$\left\{ \begin{gathered} E(s_{k} ) = \frac{k(k - 1)}{4} \hfill \\ {\text{var}} (s_{k} ) = \frac{k(k - 1)(2k + 5)}{{72}} \hfill \\ \end{gathered} \right.\;k = 2,3, \ldots ,n$$

$UF_{i}$ abides by the standard normal distribution. According to the reverse order of time series and repeating the above process, making ${UB}_{k}$ as follows:

5 $$UB_{k} = - UF_{k} (k = n,n - 1, \ldots ,1),\;UB_{1} = 0$$

then we can obtain a series of ${UB}_{k}$(k = 1,2, $\ldots$, n).

### Wavelet periodic analysis

To show the multi-level variation rules of hydrological phenomena, wavelet analysis primarily uses the localization characteristics of the wavelet function in the time domain and frequency domain^53–56^. The wavelet function is expressed as follows:

6 $$\mathop \smallint \limits_{ - \infty }^{ + \infty } \psi \left( t \right)dt = 0, \psi \left( t \right) \in L^{2} \left( R \right)$$

where ψ(t) is the wavelet basis function, and its function system is expressed as follows:

7 $$\psi_{a,b} \left( t \right) = \left| a \right|^{ - 1/2} \psi \left( {\frac{t - b}{a}} \right)a,b \in R, a \ne 0$$

where ψa,b(t) is the daughter wavelet; a is the scale factor, which reflects the period length of the wavelet function; b is the displacement factor, which reflects the shift of the wavelet function in time.

If ψ_a,b_(t) is the subwavelet given by (7), for a wavelet function that satisfies certain conditions ψ(t), L^2^(R) means it is defined on the real axis, as a measurable square integrable function on the real axis. The wavelet transform of f(t) ∈ L^2^(R) is as follows:

8 $$w_{f} \left( {a,b} \right) = \left| a \right|^{{ - \frac{1}{2}}} \mathop \smallint \limits_{ - \infty }^{\infty } f\left( t \right)\overline{\Psi }\left( {\frac{t - b}{a}} \right)dt a,b \in R and a \ne 0$$

where ψ_a,b_(t) is the wavelet transform coefficients; f(t) is a square integrable function; a is an extension scale; b is a translation parameter; $\overline{\Psi }\left( {\frac{t - b}{a}} \right)$ is the complex conjugate function of $\psi \left( {\frac{t - b}{a}} \right)$.

The square value of the wavelet coefficient is integrated in the b domain, and the wavelet variance is obtained, that is:

9 $$Var\left( a \right) = \mathop \smallint \limits_{ - \infty }^{\infty } \left| {W_{f} \left( {a,b} \right)} \right|^{2} ab$$

### Spatial analysis

Using the spatial center calculating tool in ArcGIS 10.5, the spatial center of precipitation of the arid region was obtained to study the spatial movement change characteristics of the arid region over the years. By calculating the percentage of precipitation and area anomalies between El Niño years and La Niña years, the impact of ENSO on the spatial movement of arid region was analyzed.

**Table 1:** El Niño Years and La Niña Years from 1961 to 2016.

	1	2	3	4	5	6	7	8	9	10	11	12
El Niño year	1963	1965	1969	1972	1982	1987	1991	1997	2002	2004	2009	2015
Niño 3.4 index	1.125	1.65	0.65	1.475	1.375	1.575	0.675	1.975	0.975	0.625	0.70	2.00
La Niña year	1964	1970	1971	1973	1975	1985	1988	1999	2000	2007	2010	2011
Niño 3.4 index	− 0.725	− 0.725	− 0.825	− 1.40	− 1.275	− 0.60	− 1.275	− 1.175	− 0.55	− 0.95	− 1.375	− 0.725

## Supplementary Information

Supplementary Information 1.

Supplementary Information 2.

## Data Availability

The datasets produced throughout and/or analyzed throughout the current evaluation are available from the corresponding author on reasonable request. The daily meteorological data are offered by National Earth System Science Data Center of China ( http://www.geodata.cn/ ) and China Meteorological Administrator's website ( http://cdc.cma.gov.cn/ ) in 10 January 2021.
